# The *Neurospora crassa* PCL-1 cyclin is a PHO85-1 (PGOV) kinase partner that directs the complex to glycogen metabolism and is involved in calcium metabolism regulation

**DOI:** 10.3389/fmicb.2022.1078972

**Published:** 2022-12-22

**Authors:** Jonatas Erick Maimoni Campanella, Thiago de Souza Candido, Luiz Carlos Bertucci Barbosa, Antoniel Augusto Severo Gomes, Carla Andréa Leite, Erika Silva Higashi, Paula Aboud Barbugli, Marcos Roberto de Matos Fontes, Maria Célia Bertolini

**Affiliations:** ^1^Departamento de Bioquímica e Química Orgânica, Instituto de Química, Universidade Estadual Paulista, Araraquara, São Paulo, Brazil; ^2^Departamento de Biofísica e Farmacologia, Instituto de Biociências, Universidade Estadual Paulista, Botucatu, São Paulo, Brazil; ^3^Departamento de Materiais Dentários e Prótese, Faculdade de Odontologia, Universidade Estadual Paulista, Araraquara, São Paulo, Brazil

**Keywords:** CDK/cyclin complex, protein phosphorylation, stress response, CRZ-1 transcription factor, *Neurospora crassa*

## Abstract

Cyclins are a family of proteins characterized by possessing a cyclin box domain that mediates binding to cyclin dependent kinases (CDKs) partners. In this study, the search for a partner cyclin of the PHO85-1 CDK retrieved PCL-1 an ortholog of yeast Pcls (for Pho85 cyclins) that performs functions common to Pcls belonging to different cyclin families. We show here that PCL-1, as a typical cyclin, is involved in cell cycle control and cell progression. In addition, PCL-1 regulates glycogen metabolism; Δ*pcl-1* cells accumulate higher glycogen levels than wild-type cells and the glycogen synthase (GSN) enzyme is less phosphorylated and, therefore, more active in the mutant cells. Together with PHO85-1, PCL-1 phosphorylates *in vitro* GSN at the Ser636 amino acid residue. Modeling studies identified PHO85-1 and PCL-1 as a CDK/cyclin complex, with a conserved intermolecular region stabilized by hydrophobic and polar interactions. PCL-1 is also involved in calcium and NaCl stress response. Δ*pcl-1* cells are sensitive to high NaCl concentration; on the contrary, they grow better and overexpress calcium responsive genes under high calcium chloride concentration compared to the wild-type strain. The expression of the calcium-responsive CRZ-1 transcription factor is modulated by PCL-1, and this transcription factor seems to be less phosphorylated in Δ*pcl-1* cells since exhibits nuclear location in these cells in the absence of calcium. Our results show that PCL-1 locates at different cell regions suggesting that it may determine its activity by controlling its intracellular location and reveal an interesting functional divergence between yeast and filamentous fungus cyclins.

## Introduction

Cyclins are a family of structurally related and highly conserved proteins that regulate cell cycle, transcription, and other cellular processes. Numerous cyclins have been described in the genomes of higher eukaryotes associated with various stages of the cell cycle. They can associate with proteins called cyclin dependent kinases (CDKs), a family of serine/threonine kinases, regulating their activity and determining the substrate specificity of the kinase complex. The association with CDKs and the additional regulation of CDKs by phosphorylation result in a highly intricate regulatory complex that drives the cellular events (Andrews and Measday, 1988; Noble et al., 1997; Conrad et al., 2014). In addition to their important role in controlling of cell cycle progression, CDKs also modulate transcription in response to signals being considered attractive targets for drug development (Gutierrez-Chamorro et al., 2021).

The Pho85p is a CDK from *Saccharomyces cerevisiae* that is activated by different cyclins, resulting in gene expression regulation, which affects many cellular processes depending on the cyclin partner (Carroll and O’Shea, 2002; Huang D. et al., 2007). Unlike CDKs involved in the cell cycle that require phosphorylation of a specific serine or threonine residue for maximal activation, phosphorylation of Pho85 is not required for activity (Nishizawa et al., 1999). In yeast, 10 Pho85 cyclins, referred to as Pcls (for Pho85 cyclins), have been identified, which have been grouped in two subfamilies, the Pho80 subfamily consisting of Pho80, Pcl6, Pcl7, Pcl8, and Pcl10 and the Pcl1,2 subfamily comprising the Pcl1, Pcl2, Pcl5, Pcl9, and Clg1 (Huang D. et al., 2007). The association to a particular cyclin directs Pho85 to various cellular processes, such as metabolic pathways (phosphate, carbohydrate, and lipid metabolism) and cell cycle functions (Measday et al., 1997; Smets et al., 2010; Choi et al., 2012; Prieto et al., 2020). One of the best characterized substrates for the Pcl-Pho85 complex is the yeast glycogen synthase (Gsy2p), the predominant and nutritionally regulated enzyme that catalyzes the rate limiting step in the synthesis of the storage carbohydrate glycogen. The glycogen synthase enzyme family is known to be negatively regulated by phosphorylation in multiple sites, both at the N- and C-termini, which are substrates for different kinase proteins that inactivate the enzyme preventing high glycogen accumulation (Roach et al., 2012). The Gsy2 enzyme possesses three C-terminus phosphorylation sites, and the Pho85 protein has been described as a kinase that negatively regulates glycogen accumulation by Gsy2 phosphorylation. The Pcl8 and Pcl10 cyclins were the first to be implicated in the enzyme phosphorylation, and the Pcl10-Pho85 complex was demonstrated to directly phosphorylate the enzyme (Huang et al., 1996, 1998; Wilson et al., 1999). Additionally, the Pcl6 and Pcl7 cyclins have also been reported to be involved in the control of glycogen accumulation by the Pho85 protein kinase (Wang et al., 2001a).

An important issue is understanding the structural basis of how Pcls target Pho85 to different substrates and the regulatory mechanisms involved in identifying the activating signals for each Pcl-Pho85 complex. The crystal structures of the Pho85-Pho80 (Huang K. et al., 2007) and Pho85-Pcl10 (Zheng and Quiocho, 2013) complexes were reported, and revealed important information for understanding substrate recognition and how the complex makes unnecessary the phosphorylation of the Pho85 activation loop, which is required for full activation of CDK-cyclin complex acting in cell cycle (Nishizawa et al., 1999).

The fungus *Neurospora crassa* has been widely used as a model organism for fundamental aspects of eukaryotic biology, and we have been studying the main regulatory molecular mechanisms that work together to maintain proper glycogen levels in this fungus. Using a collection of *N. crassa* protein kinase mutant strains, we searched for protein kinases that regulate glycogen metabolism, and the ORF NCU07580 was identified as a putative kinase involved in glycogen metabolism control. The heterokaryon knockout strain exhibits higher glycogen levels and less phosphorylated glycogen synthase enzyme; therefore, an enzyme more active than the one from wild-type strain (Candido et al., 2014). This protein is annotated in the fungus database as a cyclin-dependent protein kinase, and was formerly named as PGOV (Peleg et al., 1996). Based on its high identity to the *S. cerevisiae* Pho85 protein, we renamed here as PHO85-1, according to *N. crassa* nomenclature (Perkins et al., 1982). As previously mentioned, Pho85 CDK requires different cyclins for activation, and, in yeast, the Pcl6, 7, 8, and 10 cyclins are described as partners proteins that target the kinase to glycogen substrate. To identify the *N. crassa* partner cyclin that directs PHO85-1 to glycogen substrate, the four yeast cyclins were used as queries and only the ORF NCU08772 was retrieved. The encoded protein is annotated as nuclear division-60 and is named here as PCL-1 as it is the first PCL described and biochemically characterized in *N. crassa*.

We demonstrated here that the *N. crassa* PCL-1 is a real cyclin based on its structural features and that directs PHO85-1 kinase to glycogen metabolism. The Δ*pcl-1* strain accumulates higher glycogen levels than the wild-type strain and the glycogen synthase (GSN) enzyme is less phosphorylated and, therefore, more active in the mutant strain. PCL-1, together with PHO85-1, phosphorylates *in vitro* GSN, and the Ser636 residue was identified as the PHO85-1/PCL-1 complex phosphorylation site. In addition, we demonstrate that the cyclin is involved in cellular functions not only related to carbohydrate metabolism regulation. The Δ*pcl-1* cells exhibit defects in germination, the protein influences the progressing of cell division, and is involved in calcium metabolism regulation. A complemented strain was constructed, and the wild-type phenotypes were restored indicating that the defects observed are due to the protein lacking. The *N. crassa* PCL-1 multifunctional cyclin characterized in this work reveals functions common to cyclins belonging to different classes in *S. cerevisiae*, an organism that synthesizes various cyclins targeting the Pho85 kinase to different cellular functions.

## Materials and methods

### *Neurospora crassa* strains and culture conditions

The *N. crassa* FGSC#2489 wild-type strain and the FGSC#9718 (*mat a mus-51*:*bar*), FGSC#9568 (*mat a mus-52*:*hph*), FGSC#18393 (a NCU08772:*hyg*), FGSC#18394 (A NCU08772:*hyg*), FGSC#6103 (A *his-3*), FGSC#18932 (NCU06687:*hyg*), and FGSC#9518 (A, *his-3^+^*:*Pccg-1*-hH1^+^-*sfgfp*^+^) mutant strains were purchased from the Fungal Genetics Stock Center (FGSC, University of Missouri, Kansas City, MO, USA)^1^ (McCluskey, 2003). The strain Δ*pcl-1 hH1-sfgfp* was generated by crossing the FGSC#18393 and #9518 strains in 0.1 × SC Westergard medium (Westergaard and Mitchell, 1947) containing 0.5% sucrose. Gene knockout in all mutant strains was confirmed by PCR using specific oligonucleotides (Supplementary Table 1) by comparing to the amplification in the genomic DNA of the wild-type strain. The strains were maintained on solid Vogel’s minimal (VM) medium, pH 5.8 (Vogel, 1956) containing 2% sucrose at 30°C. Conidia from 10-day old cultures were suspended in sterile water, counted, and used in the experiments.

For stress analyses in plates, 5 μl of a 2 × 10^7^ conidia per mL suspension from wild-type, Δ*pcl* and Δ*pcl-1 pcl-1^+^* (complemented) strains were inoculated on Petri dishes in solid VM medium containing 2% sucrose and either NaCl or sorbitol for osmotic stress assays, or H_2_O_2_ for oxidative stress assays, or Congo Red (CR) for plasma membrane/cell wall stress assay, or calcium chloride for calcium sensitivity assay, or at pH 7.8 for alkaline stress assay. Cultures were incubated at 30°C, and the colony diameter was measured after 24 h.

### Construction of the Δ*pcl-1 pcl-1^+^* complemented strain

For complementation, the FGSC#18393 strain (Δ*pcl-1*:*hyg*) was crossed with the FGSC#6103 strain (*his-3*) to generate the Δ*pcl-1 his-3* double mutant strain. A DNA fragment of 1,156 bp was amplified by PCR with the primers 8772sfGFP-F and 8772sfGFP-R (Supplementary Table 1) using genomic DNA from the wild-type strain as template. The 8772sfGFP-R oligonucleotide contains the sequence that codifies for 6-Gly between the nucleotide sequences encoding sfGFP and the PCL-1 proteins. PCR was performed using a Phusion High-Fidelity PCR kit (Finzymes), and the DNA fragment was purified with a QIAquick Gel Extraction Kit (Qiagen, Hilden, Germany) according to the manufacturer’s instructions. The purified DNA fragment was cloned into the *Pac*I and *Xba*I sites of the pTSL91-A plasmid (a donation from N. L. Glass, University of California at Berkeley, Berkeley, CA, USA), generating the pTSL91A-*pcl-1* plasmid. This plasmid allows the constitutive expression of the C-terminus PCL-1-sfGFP fusion protein, as the *ccg-1* promoter drives the *pcl-1* gene expression. To express the protein under the control of native promoter, the *ccg-1* promoter was removed with the *Not*I and *Xba*I enzymes and replaced by the *pcl-1* promoter (1,500 bp) amplified by PCR using the np8772-F and np8772-R oligonucleotides (Supplementary Table 1), generating the construction pTSL91A-P*pcl-1-pcl1*-*sfgfp*. Plasmid constructions were used to transform by electroporation competent conidia from the recipient Δ*pcl-1 his-3* double mutant strain. The transformants were selected on VM media containing hygromicin (200 μg per mL) without histidine and confirmed by PCR using the primers np8772-F and 8772sfGFP-R (Supplementary Table 1). The progeny was analyzed by fluorescence microscopy, and the complemented strain (Δ*pcl-1 pcl-1^+^*) was evaluated in different experiments.

### Germination and cell cycle progression assays

The analyses were performed in submerged cultures, and 2 × 10^7^ conidia per mL (final concentration) from WT, Δ*pcl* and Δ*pcl-1 pcl-1^+^* strains were inoculated into flasks containing 30 mL of liquid VM medium plus 2% sucrose, pH 5.8, 30°C, 150 rpm. Aliquots were removed every 2 h, and the conidia in germination were counted. Germination was also analyzed in conidia inoculated onto coverslips covered with liquid VM plus 2% sucrose, pH 5.8 and incubated at 30°C for different times. For nuclei analysis, cells were fixed [1% phosphate buffered saline (PBS), 3.7% formaldehyde, 0.1% Triton X-100], washed twice with PBS and stained with 100 μL DAPI (4′,6-diamidino-2-phenylindole, 0.5 mg per mL in DMSO) for 5 min. DAPI fluorescence was visualized using a fluorescence microscope with excitation and emission wavelengths of 358 nm and 463 nm, respectively. Images of conidia germination were captured using an AXIO Imager.A2 Zeiss microscope, at a magnification of 1000 X, coupled to an AxioCam MRm camera and processed with the using the AxioVision software.

The cell cycle progression of the Δ*pcl-1 hH1-sfgfp* strain was analyzed by inoculating 2 × 10^7^ conidia per mL (final concentration) into flasks containing 30 mL of liquid VM medium plus 2% sucrose, pH 5.8, 30°C, 150 rpm. The FGSC#9518 (A, *his-3^+^*:*Pccg-1*-hH1^+^-*sfgfp*^+^) strain was used as control. Aliquots were removed every 2 h, and the GFP fluorescence was analyzed in the AXIO Imager.A2 Zeiss fluorescence microscope coupled to an AxioCam MRm camera and processed using the AxioVision 4.8.2 software with excitation and emission wavelengths of 490 and 525 nm, respectively. Images were captured at different times, and six images from each strain were used for quantification of the nuclei in the different phases of mitosis (approximately 200) (Hong et al., 2014).

### Quantification of glycogen and glycogen synthase activity

Glycogen and glycogen synthase activity were quantified in mycelia pads grown by inoculating 10^7^ conidia per mL into 500 mL of liquid VM, 250 rpm at 30°C. Mycelia samples were collected every 12 h and frozen in liquid nitrogen. Mycelia pads were extracted in lysis buffer (50 mM Tris-HCl, pH 8.0, 50 mM NaF, 1 mM EDTA, 0.5 mM PMSF, 0.1 mM TCLK, 25 mM benzamidine, and 1 μg per mL of each pepstatin and aprotinin). Cellular extracts were clarified by centrifugation at 3,000 X *g*, 10 min, 4°C, and the supernatants were used for glycogen, glycogen synthase activity, and protein quantification. Glycogen quantification was performed after precipitation with cold ethanol and digestion with α-amylase and amyloglucosidase (Freitas et al., 2010). Free glucose was quantified with a glucose oxidase kit (Labtest, Brazil), and glycogen concentration was normalized to the total protein concentration. Glycogen synthase activity was measured in the presence and in the absence of G6P (glucose-6-phosphate, 7.2 mM final concentration) and normalized to the total protein concentration (Thomas et al., 1968). Total protein was quantified using BSA as standard (Hartree, 1972).

### GSN phosphorylation analysis by 2-DE

Glycogen synthase phosphorylation was analyzed by two-dimensional electrophoresis in mycelial pads from the wild-type and Δ*pcl-1* strains grown for 24 h (Candido et al., 2014). Briefly, total protein was fractionated by IEF using the pH 4–7 gradient Immobiline™ DryStrip (13 cm, linear, GE Healthcare, Chicago, USA) in an Ettan IPGphor 3 system (GE Healthcare, Chicago, USA), according to the manufacturer’s instructions. IEF was carried out at 50 μA per strip at 20°C, using the following steps: 100 V (10 h), 500 V (500 Vh), 1,000 V (750 Vh), 8,000 V (11,325 Vh), and 8,000 V (5,067 Vh). After electrofocusing, the strips were equilibrated and subjected to a 9% SDS-PAGE, using a Hoefer™ SE600 system (GE Healthcare, Chicago, USA). To analyze the GSN phosphorylation status, proteins were transferred to nitrocellulose membrane after 2-DE and blotted with anti-GSN antibody raised in rabbit. Blots were subsequently probed with HRP-conjugated secondary antibodies (Sigma) and developed with luminol reagent. As a control of phosphorylation, total protein from the wild-type strain was treated with λ-protein phosphatase according to the suppliers’ protocol (#P0753S, New England Biolabs, Ipswich, USA).

### Production and purification of recombinant proteins

The *pcl-1* cDNA (ORF NCU08772, 1,110 bp) was amplified by RT-PCR with the oligonucleotides Nc8772-F/Nc8772-R (Supplementary Table 1) using cDNA synthesized from total RNA and the SuperScript III reverse transcriptase kit (Invitrogen, Waltham, USA). The DNA fragment was cloned into *Nde*I and *Bam*HI sites of pET28a expression vector leading to the pET-*pcl-1* construction for the expression of a 369 amino acids protein fused to His-tag at N-terminus. The *pho85-1* cDNA (ORF NCU07580, 1,014 bp) was amplified by RT-PCR with the oligonucleotides Nc7580-F/Nc7580-R (Supplementary Table 1) and cloned into *Nde*I and *Bam*HI sites of pET28a expression vector leading to the pET-*pho85-1* construction for the expression of a 337 amino acids protein fused to His-tag at N-terminus. The full *gsn* cDNA was amplified from the pET28a-*gsn* plasmid (Freitas et al., 2010) with the GSN-*Bam*HI-F and GSN-*Eco*RI-R oligonucleotides and subcloned into *Bam*HI and *Eco*RI sites of a modified pET28a expression vector, leading to a His-tagged SUMO fusion protein at N-terminus. Site-directed mutagenesis at serine/threonine amino acids in SUMO-GSN was performed using the QuickChange mutagenesis kit (Stratagene, San Diego, CA, USA). The S632A, S636A, T641A, and T645A single mutations were introduced with the S632A-F/R, S636A-F/R, T641A-F/R, and T645A-F/R oligonucleotides pairs, respectively. The quadruple mutant protein S632A/S636A/T641A/T645A was constructed by previously constructing the double mutant S632/636A using the S636ADM-F/R oligonucleotides and the S632A mutant cDNA as model. The double mutant cDNA was used to construct the triple mutant S632/636AT641A using the T641A-F/R oligonucleotides, which was used to construct the quadruple mutant using the T645ADM-F/R oligonucleotides. All plasmid constructions were confirmed by DNA sequencing. Recombinant proteins were produced according to Campanella et al. (2021) with slight modifications. The His-SUMO protein was also produced under the same induction conditions and used as control in the *in vitro* protein phosphorylation experiments.

### *In vitro* phosphorylation assay

For the phosphorylation assays, 25 μg of the wild-type and mutants His-SUMO-GSN proteins were incubated with 10 μg of His-PHO85-1 and 10 μg of His-PCL-1 proteins in reacting buffer (1 mM ATP, 25 mM MgCl_2_, [γ-^32^P]-ATP, activity 330 mCi/mmol, ∼1200 cpm/mmol) in 25 μL of reaction volume for 30 min at 30°C. Crude cellular extract of a *N. crassa* mutant strain (Δ*gsn*), which does not synthesize glycogen synthase, was also used as a source of protein kinases. After incubation, His-SUMO-GSN proteins were immobilized on Ni-NTA agarose beads (Qiagen, Hilden, Germany) under low agitation. The beads were collected by centrifugation, washed three times in washing buffer (50 mM Tris, pH 8.0, 30 mM imidazole, 500 mM NaCl) and suspended in elution buffer (50 mM Tris, pH 8.0, 500 mM imidazole, 500 mM NaCl). After protein elution, Laemmli buffer (Laemmli, 1970) was added, the protein samples were boiled for 5 min and separated on a 12% SDS-PAGE gel. After electrophoresis, the gel was stained with Coomassie Brilliant Blue (CBB), dried and exposed to X-ray film.

### Construction of V5- and GFP-tagged proteins

For protein expression and cellular localization, strains expressing at C-terminus either V5- or GFP-tagged CRZ-1 proteins were constructed using the Honda and Selker (2009) methodology, as previously described (Campanella et al., 2021). The pZERO-*hph-V5* and pZERO-*hph-gfp* plasmids (a donation from M. Freitag, Oregon State University, Corvallis, OR, USA) were used as templates and the splitmarkers were amplified by PCR using the CrzGly-F/R, and CrzLox-F/R oligonucleotide pairs, listed in Supplementary Table 1. The *hph-V5* and *hph-gfp* nucleotide sequences were amplified from pZERO plasmids using the 10xGly-F/LoxP-R pair (Supplementary Table 1). The PCR fragments were fused to the *hph-V5* or *hph-gfp* fragments by PCR using the CrzGly-F/Hph-R, Hph-F/CrzLox-R oligonucleotide pairs (Supplementary Table 1). Fusion DNA fragments were individually transformed into #9718 strain, and transformants were selected in the presence of hygromycin. Homokaryons were isolated by crossing with either the FGSC#2489 or FGSC#18394 strains in 0.5% Westergaard’s medium leading to the *crz*-*1*:*crz*-*1-V5* and *crz-1*:*crz-1*-*gfp* in the wild type and in the Δ*pcl-1* background strains. The segregants from each strain were confirmed by PCR using the Nc8772-F/R, CrzGly-F, V5-R, and GFP-R primers (Supplementary Table 1). *N. crassa* transformation and other molecular techniques were performed using the protocols available at the Neurospora homepage.^2^

### Gene and protein expression

For gene expression analysis, conidia from the WT and Δ*pcl-1* strains were grown in VM media containing 2% sucrose for 24 h with and without 300 mM CaCl_2_. The mycelia were harvested by filtration, washed with sterile water, and submitted to total RNA extraction (Sokolovsky et al., 1990). Total RNA (10 μg) samples were first treated with RQ1 RNase-free DNase (Promega, Madison, WI, USA) and subjected to cDNA synthesis by using SuperScript III First-Strand Synthesis kit (Invitrogen, Waltham, USA) and an oligo (dT) primer, according to manufacturer’s instructions. The cDNA libraries were subjected to RT-qPCR on a StepOnePlus™ Real Time PCR System (Applied Biosystems, Waltham, USA) using the Power SYBR^®^ Green PCR Master Mix (Applied Biosystems, Waltham, USA) and specific primers for each gene amplicon (Supplementary Table 1) as previously described (Virgilio et al., 2017). Data analysis was performed by the StepOne Software (Applied Biosystems, Waltham, USA), the comparative CT (ΔΔCT) method (Livak and Schmittgen, 2001) was used to estimate the Log2 fold expression. At least three biological replicates, with three experimental replicates each were performed, and reactions with no template were used as negative control. The PCR products were subjected to melting curves analysis to verify the presence of a single amplicon. All reaction efficiencies varied from 94 to 100% and the results were expressed relative to the expression of the beta-tubulin reference gene (β-*tub*-2 gene, NCU04054).

For protein expression, conidia from *crz*-*1*:*crz-1-V5* and Δ*pcl-1 crz-1:crz-1-V5* strains were grown for 24 h in VM containing 2% sucrose at 30°C and 180 rpm. After 24 h, the mycelia were harvested by filtration and divided in samples. One sample was frozen in liquid nitrogen and stored at −80°C until use (control sample). The remaining samples were individually transferred into 150 ml of fresh VM liquid medium containing either 0.5% sucrose and 10 or 300 mM CaCl_2_ for up to 60 min at 30°C and 180 rpm. The stressed mycelia were harvested, washed with sterile water and frozen in liquid nitrogen. Protein extraction was performed according to Campanella et al. (2021). For analysis of phosphorylated proteins 1 mM NaF, 1 mM sodium orthovanadate, and 1x EDTA-Free Phosphatase Inhibitor Cocktail (Sigma) was added to the lysis buffer. For protein phosphatase treatment, samples prepared in lysis buffer without phosphatase inhibitor were added of 2 mM MnCl_2_ and treated with 400 U of λ-protein phosphatase (#P0753S, New England Biolabs, Ipswich, USA) for 40 min at 30°C followed by boiling for 5 min in 1 X Laemmli sample buffer (Laemmli, 1970). Separation of CRZ-1-V5 was accomplished in 4–15% Mini-PROTEAN^®^ TGX™ Precast Gels using 30 μg of total protein (Hartree, 1972). The proteins were electro-transferred to nitrocellulose blotting membrane (BioRad, Hercules, USA) and probed with a monoclonal anti-V5 antibody (Invitrogen, Waltham, USA). Blots were subsequently probed with HRP-conjugated secondary antibodies (Invitrogen, Waltham, USA) and developed with ECL reagent (BioRad, Hercules, USA). Images were acquired using the ChemiDoc MP Imaging System (BioRad, Hercules, USA).

### Cellular localization

To determine the cellular localization of the CRZ-1-GFP protein under calcium stress, 200 μL of a conidial suspension (2 × 10^6^ conidia per mL) from the *crz*-*1*:*crz*-*1*-*gfp* and Δ*pcl-1 crz*-*1*:*crz-1-gfp* strains were inoculated onto coverslips, covered with VM media plus 2% sucrose and incubated at 30°C for 8 h (control sample). After incubation, the coverslips were transferred to fresh VM media containing 0.5% sucrose and 300 mM CaCl_2_, and incubated for 30 min. The PCL-1 localization during germination was performed by inoculating 2 × 10^7^ conidia per mL (final concentration) of Δ*pcl-1 pcl-1^+^* complemented strain, which express the sfGFP-tagged PCL-1 under the native promoter. Coverslips were removed and protein location was analyzed every 2 h. For nuclei analysis, all samples were immediately fixed in 3.7% formaldehyde, 1% phosphate buffered saline (PBS), pH 7.0, 0.2% (v/v) Tween 80, washed twice with PBS and stained with Hoechst (10 μg per mL) for 15 min, followed by washing with PBS. Confocal Laser Scanning Microscopy (CLSM) was performed using a CARLS ZEISS LSM 800 Confocal Microscopy with a Plan Apochromat 63x/1.40 Oil DIC M27 objective. The detection parameters for all experiments were fixed at (i) nuclei analysis–hoechst—laser 405 nm: 35% 2.21 AU/79 μm, detection wavelength 400–450 nm, detection gain 894 V; (ii) CRZ-1-GFP–laser 488 nm: 35% 1.89 AU/84 μm detection wavelength 488–574 nm, detection gain 850V. The z-stack increments were 0.3 μm. The images were analyzed using the ZEN Blue 2.3.

### Molecular modeling study of PCL-1 and PHO85-1

PCL-1 and PHO-85-1 sequences were submitted to AlphaFold^3^ to obtain their respective predicted models separately and also in complex, using AlphaFold Multimer.^4^ The atomic coordinates of ATP and Mg^2+^ were retrieved from the crystallographic structure of the *S. cerevisiae* Pcl10/PHO85 complex available in Protein Data Bank (PDB ID: 4KRC)^5^ after superposition of the complexes. Then, the PCL-1/PHO85-1 complex was submitted to molecular dynamics (MD) simulations using GROMACS v.2020.4 (Abraham et al., 2015) under the CHARMM36m force field (Huang et al., 2017). The protonation state of the complex was determined according to the PROPKA3 web server (Olsson et al., 2011), setting the residue His290 of PCL-1 as positively charged. The complex was placed in a rhombic dodecahedric box of 12 Å distant from the farthest atom in XYZ directions. The system was then solvated and equilibrated with 0.15 M of NaCl, adding two Cl^–^ to obtain the net charge of zero. Following, the system was minimized using the Steepest Descent algorithm until reaching an energy gradient below 100 kJ/mol/nm^2^. An NVT ensemble was applied generating the initial velocities randomly following a Maxwell–Boltzman distribution at 300 K during 1 ns, using the V-Rescale thermostat (Bussi et al., 2007) with a time constant of 0.1 ps. An NPT ensemble of 1 ns was then applied, defining 1 bar as reference pressure, using the Berendsen barostat (Eslami et al., 2010) with a time constant of 1.0 ps. These steps were performed restraining the backbone atoms of both proteins, ATP heavy atoms and Mg^2+^ under a force constant 1.000 kJ/mol/nm^2^. An unconstrained MD of 300 ns was applied using the Nose–Hoover thermostat (Hoover, 1985) and Parrinello–Rahman barostat (Parrinello and Rahman, 1981), using a time constant of 0.5 and 5.0 ps, respectively. Non-bonded interactions were calculated considering atoms within 10 Å using the PME method, with a switching force function between 10 and 12 Å. Three independent replicas were performed collecting frames every 20 ps. After processing the PBC conditions of all frames, RMSD and RMSF calculations were performed for backbone atoms of the complex, using GROMACS built-in tools. Further, the prevalence of contacts for each residue of PCL-1 or PHO85-1 was measured considering heavy atoms of pairs of residues within a 3.5 Å cutoff, using a homemade tcl script implemented in VMD (Humphrey et al., 1996).

## Results

### Molecular cloning of the *Neurospora crassa pcl-1* and molecular modeling of the PCL-1

**FIGURE 1:**
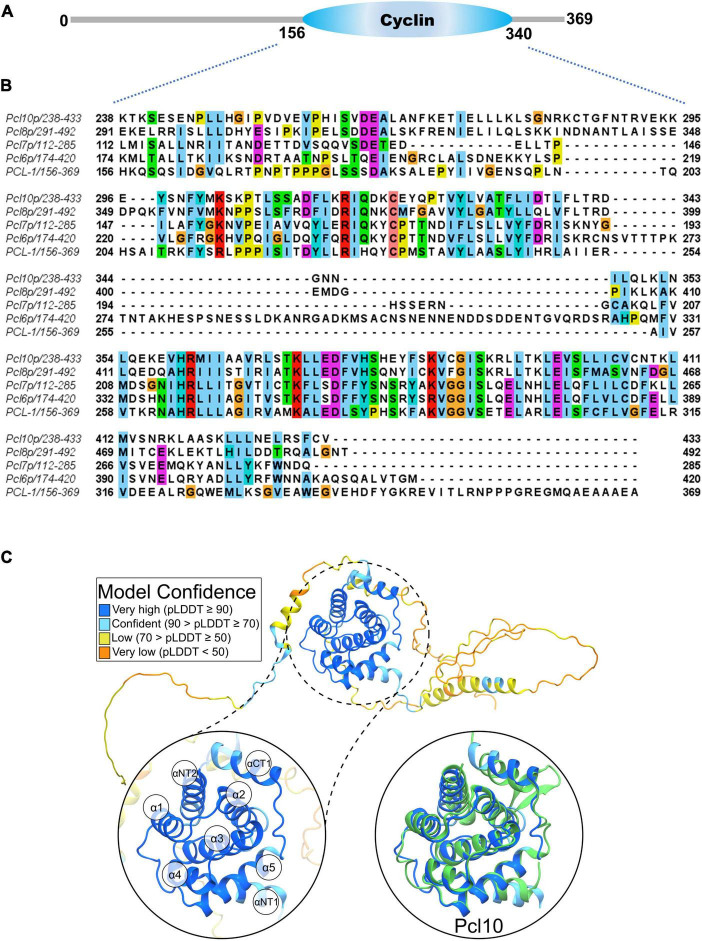
The protein encoded by ORF NCU08772 exhibits similarities to *S. cerevisiae* Pcl cyclins and possesses a typical cyclin box domain. **(A)** Conserved cyclin domain in the NCU08772 product according to PFAM. **(B)** Sequence alignment between the polypeptide sequences of the cyclin domain identified in the ORF NCU08772 product (156–340) and Pcl cyclins from *S. cerevisiae* (Pcl6p, Pcl7p, Pcl8p, and Pcl10p). Each amino acid is represented by different colors in the conservative regions. Sequence alignment was performed using ClustalW2 and the identical and conservative amino acids were identified using Jalview Version 2 (Waterhouse et al., 2009). **(C)** PCL-1 model predicted by AlphaFold, emphasizing the conserved helices of the CBD and the superposition to Pcl10 shown in green (zoom-in-view panels). Model confidence of PCL-1 residues are defined as very high, confident, low, and very low confidences shown as blue, cyan, yellow, and orange, respectively.

In a previous study, we identified the ortholog of the *S. cerevisiae* Pho85p cyclin-dependent protein kinase, the ORF NCU07580 product, as a regulatory protein of the glycogen metabolism (Candido et al., 2014). In *S. cerevisiae*, the Pho85p protein kinase together with Pcl6p, Pcl7p, Pcl8p, and Pcl10p (for Pho85p cyclins) cyclins has been described as a protein that controls glycogen metabolism by controlling glycogen synthase (Gsy2p) phosphorylation (Wilson et al., 1999; Wang et al., 2001b). To identify the partner cyclin of PHO85-1 involved in the glycogen metabolism control in *N. crassa*, a BLASTp search in the *N. crassa* genome database^6^ using as queries the *S. cerevisiae* cyclins, retrieved a homolog protein encoded by the ORF NCU08772, which shares an average of 24% identity to the yeast cyclins mainly in the cyclin domain (Figure 1A). The predicted protein is annotated in the fungus database as nuclear division-60, and is named here as PCL-1 consistent with the *N. crassa* nomenclature (Perkins et al., 1982) and considering that it is the first PCL described in *N. crassa*. PCL-1 is a non-essential protein, and the Δ*pcl-1* mutant strain exhibits wild-type phenotypes suggesting that it is not involved in processes related to growth and development (results not shown).

PCL-1 is composed of 369 amino acid residues and contains a characteristic cyclin domain encompassing the amino acid region from 156 to 340 (Figure 1A; Gibson et al., 1994). Sequence alignment of PCL-1 with the orthologous Pcls from yeast (Figure 1B) showed that they share a C-terminus conserved region only in the amino acid sequence corresponding to the cyclin box domain (CBD, 24% identity and e-value 3.10^–14^ compared with Pcl10). This region shows high divergence regarding the conserved amino acid residues, there are large insertions and only a few residues are identical in all proteins.

To confirm if PCL-1 has a folding characteristic of a cyclin, its structure was predicted using AlphaFold. As shown in Figure 1C, PLC-1 model presents a globular region formed by the arrangement of different α-helices predicted with high confidence. Such a region corresponds to a classical cyclin domain (Tatum and Endicott, 2020), which is composed of two N-terminal helices (αNT1 and αNT2) followed by five helices (α1–α5) and one C-terminal helix (αCT1). Furthermore, PCL-1 was superposed to Pcl10 (Zheng and Quiocho, 2013) to corroborate the presence of such a cyclin-like domain, evidenced by the structural homology of all α-helices described above with Cα atoms RMSD value of only 0.63 Å.

### PCL-1 controls glycogen levels by affecting the glycogen synthase phosphorylation status

**FIGURE 2:**
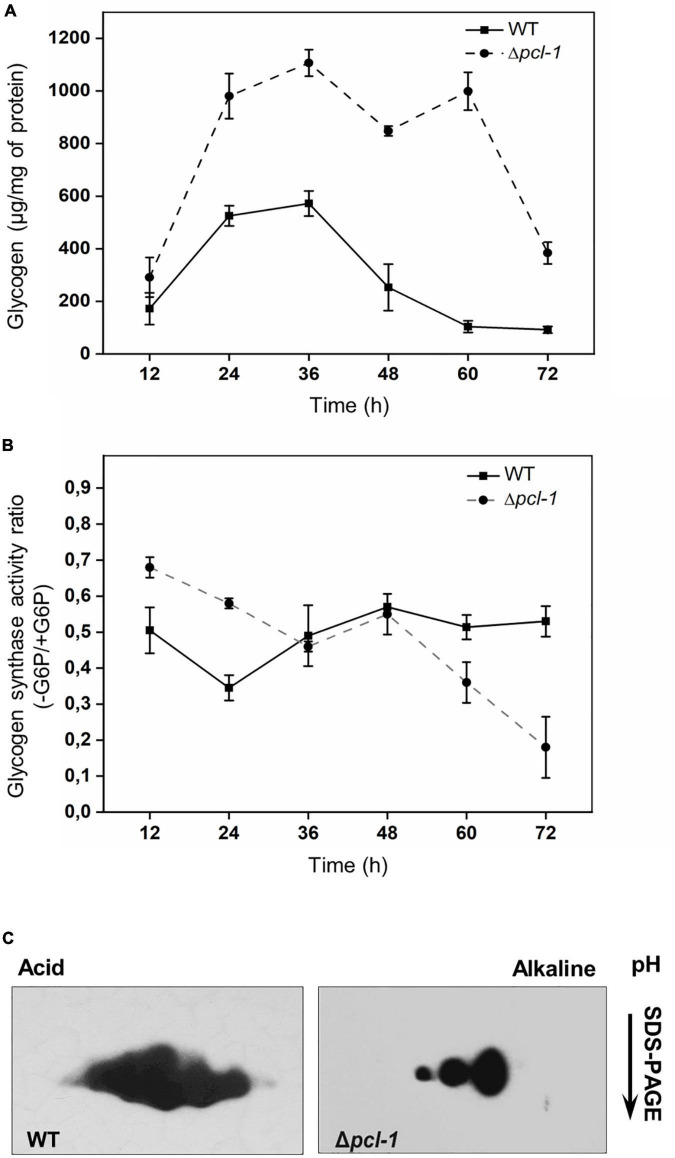
The Δ*pcl-1* strain shows increased glycogen levels and GSN activity ratio during growth and GSN is less phosphorylated in Δ*pcl-1* strain. Conidia from wild-type and mutant strains were inoculated into liquid VM, and samples were harvested every 12 h. **(A)** Glycogen accumulation was assayed by digestion with amyloglucosidase and α-amylase after ethanol precipitation. Free glucose was quantified using the glucose PAP kit (Labtest, Brazil). **(B)** Quantification of GSN activity was performed in the same extracts used for glycogen quantification. Activity was quantified in the presence and absence of G6P (glucose-6-phosphate). Error bars represent the standard deviations derived from three independent experiments. **(C)** A zoom-in view of GSN in the 2D gel images. GSN phosphorylation was analyzed in the wild-type and Δ*pcl-1* strains grown for 24 h using 2D-PAGE gels followed by Western blot with anti-GSN antibody raised in rabbits. GSN is an 80 kDa protein with a theoretical isoelectric pH of 6.0.

We investigated if PCL-1 is involved in the *N. crassa* glycogen metabolism control similar to the *S. cerevisiae* Pcl6p, Pcl7p, Pcl8p, and Pcl10p counterparts. Glycogen quantification in the Δ*pcl-1* strain at different times of vegetative growth revealed that the mutant strain accumulates higher levels of glycogen, approximately twice more as much as the wild-type strain (Figure 2A). We further quantified the glycogen synthase (GSN) phosphorylation status as an attempt to correlate the enzyme phosphorylation with the glycogen stored by the mutant strain. Phosphorylation inhibits glycogen synthase activity (Téllez-Iñón et al., 1969). Enzymatic activity was quantified in the same mycelial pads used for glycogen quantification in the presence and in the absence of glucose-6-phosphate (G6P), the glycogen synthase allosteric modulator. The −/+ G6P activity ratio is considered as an index of phosphorylation, with higher levels being correlated with lower phosphorylation and thus, higher activity. The activity ratio in the Δ*pcl-1* strain was higher than that in the wild-type strain at the initial growth times, suggesting that GSN is less phosphorylated in the mutant strain (Figure 2B). This result may explain the higher glycogen levels observed in the mutant strain under the same growth condition. As the −/+ G6P activity ratio correlates with different isoforms of phosphorylated GSN, we decided to investigate the phosphorylated forms of GSN. For that, 24 h mycelial pads from the wild-type and mutant strain were analyzed by 2D-gels followed by Western blot using anti GSN antibody (Figure 2C). Five GSN differentially phosphorylated isoforms were observed in the wild-type strain, and treatment with protein phosphatase reduces to two isoforms, as we previously demonstrated (Candido et al., 2014). Only three GSN phosphorylated isoforms were visualized in the Δ*pcl-1* strain, suggesting that GSN is less phosphorylated in this strain and that PCL-1 influences the phosphorylation status of GSN.

### PCL-1 together with the PHO85-1 protein kinase phosphorylates *in vitro* GSN

As mentioned, in *S. cerevisiae*, the serine/threonine Pho85p protein kinase, together with Pcls, phosphorylates Gsy2p (Huang et al., 1996, 1998; Wilson et al., 1999). In addition, we previously identified the ORF NCU07580 product, the ortholog of the *S. cerevisiae* Pho85p, as a kinase protein regulating glycogen metabolism (Candido et al., 2014). This protein is annotated in the *N. crassa* database as a cyclin-dependent protein kinase, and based on the high identity (64%) between the *N. crassa* and yeast kinase proteins we named the NCU07580 product as PHO85-1, according to the *N. crassa* nomenclature. The *N. crassa* protein exhibits the PSTAIRE and the glycine-rich domains (Supplementary Figure 1), which characterize CDKs (Pines, 1995; Nishizawa et al., 1999), as well as the T-loop region encompassing the threonine residue (Thr164 in PHO85-1), which needs to be phosphorylated for activation in CDKs other than Pho85p (Nishizawa et al., 1999). Using an *in vitro* GSN phosphorylation assay, we investigated whether PCL-1 would be the PHO85-1 partner cyclin that directs the kinase to glycogen metabolism. For this, both His-tagged recombinant proteins (His-PCL-1 and His-PHO85-1) were produced in *E. coli* and partially purified by affinity chromatography. The His-tagged SUMO-fused GSN wild-type and mutant proteins were also produced in *E. coli* and partially purified by affinity chromatography.

**FIGURE 3:**
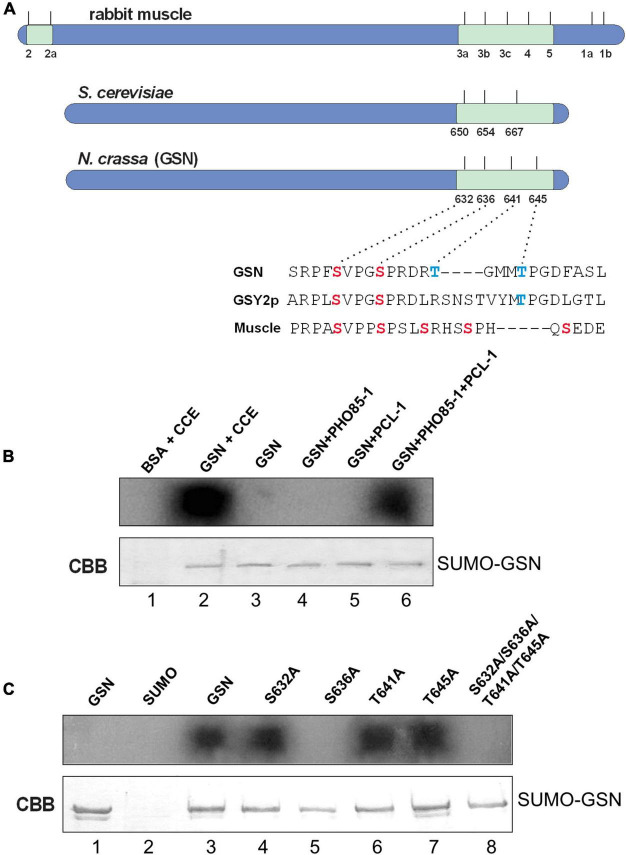
The recombinant PHO85-1/PCL-1 complex phosphorylates GSN *in vitro* at Ser636. **(A)** Characteristics of the glycogen synthase phosphorylation sites. General architecture of the rabbit muscle GS compared to the *S. cerevisiae* Gsy2p and the *N. crassa* GSN. The Ser and Thr amino acid residues are shown in red and blue, respectively. **(B,C)** Commercial BSA and the recombinant His_6_-SUMO, His_6_-PCL-1, His_6_-PHO85-1, and His_6_-SUMO-GSN wild-type and mutant proteins at different phosphorylation sites (S632A, S636A, T641A, T645A, and S632A/S636A/T641A/T645A) expressed in *E. coli* and purified were assayed for *in vitro* phosphorylation. **(B)** Phosphorylation reaction using either the His_6_-PHO85-1/His_6_-PCL-1 complex or crude cellular extract (CCE) from the Δ*gsn* strain as a source of protein kinase. Lanes 1 and 2, phosphorylation of BSA and His_6_-SUMO-GSN, respectively, using CCE. Lanes 3–6, His_6_-SUMO-GSN without protein kinase (lane 3), and in the presence of His_6_-PHO85-1 protein kinase (lane 4), His_6_-PCL-1 cyclin (lane 5), and His_6_-PHO85-1/His_6_-PCL-1 complex (lane 6). **(C)** Phosphorylation reaction using the His_6_-PHO85-1/His_6_-PCL-1 complex. Lane 1, His_6_-SUMO-GSN without protein kinase. Lanes 2–8, His_6_-SUMO (lane 2, negative control), lanes 3–8, His_6_-SUMO-GSN wild-type and mutant proteins in the presence of His_6_-PHO85-1/His_6_-PCL-1 complex. CBB, Coomassie brilliant blue.

Based on an amino acid sequence alignment with the rabbit muscle (Gys1) and the *S. cerevisiae* (Gsy2p) glycogen synthases, we identified four C-terminus putative phosphorylation sites in GSN, the Ser632, Ser636, Thr641, and Thr645 amino acid residues (Figure 3A). Like the yeast enzyme, the *N. crassa* enzyme lacks the N-terminus phosphorylation sites. We next investigate whether PCL-1 could be a partner cyclin of the PHO85-1 protein kinase in the *in vitro* GSN phosphorylation, and to identify the amino acid residue target of phosphorylation by the PHO85-1/PCL-1 complex. Site-directed mutagenesis was used to introduce point mutations in the His-SUMO-GSN by replacing the putative serine and threonine phosphorylation residues with alanine, generating the single S632A, S636A, T641A, and T645A and the quadruple S632A/S636A/T641A/T645A mutant proteins. We first analyzed the wild-type His-SUMO-GSN phosphorylation using as source of protein kinases the crude cellular extract from a knockout strain that does not synthesize GSN (FGSC#18932, *gsn:hyg*). BSA was used as a negative control in the assay. We observed that GSN was phosphorylated *in vitro* by the kinases present in the cellular extract (Figure 3B, lane 2). In addition, GSN was phosphorylated only when the PHO85-1 protein kinase and the PCL-1 cyclin were combined in the reaction mixture (Figure 3B, lane 6). This result confirms that PHO85-1 is the *N. crassa* ortholog of the yeast Pho85p protein, and that PCL-1 acts as a partner cyclin of the PHO85-1 protein kinase in the GSN phosphorylation. No phosphorylation was observed in the absence of either the cyclin (Figure 3B, lane 4) or the protein kinase (Figure 3B, lane 5). We further assayed the phosphorylation of the GSN mutants by the PHO85-1/PCL-1 complex. The GSN wild-type protein incubated in absence of the complex (Figure 3C, lane 1) and the recombinant SUMO protein (Figure 3C, lane 2) were used as negative controls. We observed that only the GSNS636A mutant was not phosphorylated *in vitro*, indicating that the Ser636 amino acid residue is the amino acid residue target of phosphorylation by the kinase/cyclin protein complex (Figure 3C, lane 5). No phosphorylation was observed when the quadruple mutant GSN (S632A/S636A/T641A/T645A) was used as substrate, indicating that no additional sites are target of phosphorylation by the PHO85-1/PCL-1 complex (Figure 3C, lane 8).

### The PSTAIRE helix of PHO85-1 is predicted with high confidence only when in complex to PCL-1

As shown in Figure 3, the PHO85-1 kinase activity depends on the presence of PCL-1, suggesting that these proteins interact to result in a functional CDK/cyclin complex. Therefore, the structure of PHO85-1 was predicted using AlphaFold as previously applied to PCL-1. Five models of PHO85-1 were generated, which converged to only two conformations (Supplementary Figure 2, models 1 and 2). In general, the predicted PHO85-1 structures presented high confidence, except for the PSTAIRE helix and the T-loop. Interestingly, these regions were predicted with high confidence using AlphaFold Multimer (Supplementary Figure 2, model 3) only when PCL-1 was present, suggesting that these regions may assume stable conformations only when in complex with their partner, resulting in a salt-bridge between the Lys39 and Glu56 residues of PHO85-1.

**FIGURE 4:**
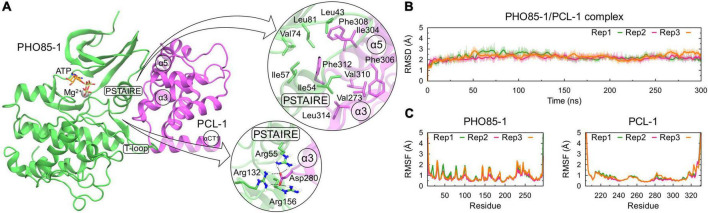
MD simulation of the PHO85-1/PCL-1 complex predicted by AlphaFold Multimer. **(A)** Initial conformation of the complex with PHO85-1 and PCL-1 shown as green and purple, respectively, highlightning important regions such as PSTAIRE and T-loop of PHO85-1, and α3, α5, and αCT1 of PCL-1. ATP and Mg^2+^ are considered presented as orange sticks and silver sphere, respectively. Salt-bridge and hydrophobic interactions between the complex are shown in detail in zoom-in-view panels. **(B)** RMSD of backbone atoms of the PHO85-1/PCL-1 complex and **(C)** RMSF of backbone atoms of PHO85-1 and PCL-1 are shown in the left and right panels, respectively. Values are shown for each replica separately.

Figure 4A shows the predicted PHO85-1/PCL-1 complex, considering the presence of ATP and Mg^2+^ molecules (see Section “Materials and methods”), which was further submitted to MD simulations to verify its structural stability and to determine the main contacts between them. Some important regions at the interface of the complex are shown, such as the PSTAIRE helix and the T-loop of PHO85-1 and the helices α3, α5, and αCT1 of PCL-1. When analyzing these regions in detail, a salt-bridge is observed between the residue Asp280, located in the α3 helix of PCL-1, and three Arginine residues of PHO85-1, named Arg55 (PSTAIRE helix) and Arg132 and Arg156 (T-loop). Another important region found to stabilize the complex was determined by hydrophobic interactions between the PSTAIRE helix residues, such as Ile54, and the PCL-1 residues placed in the α3 and α5 helices, such as Phe312 and Val273 (Figure 4A, zoom-in-view panels).

Molecular dynamics (MD) simulations of the PHO85-1/PCL-1 complex showed considerable stability and reproducibility in all three replicas, presenting a root-mean-square deviation (RMSD) average value of 2.2 ± 0.49 Å (Figure 4B) and root mean-square fluctuation (RMSF) values for each protein below 3 Å in every replica (Figure 4C), except for the first N- and last C-terminal residues. Considering the whole amount of data, it was possible to determine the prevalence of contacts of the PHO85-1/PCL-1 interface. The salt-bridge led by Asp280 residue of PCL-1 is strongly stable, presenting contacts to Arg55, Arg132, and Arg156 during 74.83, 99, 70, and 75.02% of the time. Asp280 is surrounded by these three Arginine residues, allowing an environment with high complementarity of charges. Moreover, a hydrophobic-rich region formed by the complex was also considered important for stabilizing the PHO85-1/PCL-1 complex (Figure 4A, zoom-in-view panels), highlighting the contacts of Phe312 (PCL-1) to Ile57 (PHO85-1) during 99.77%, and Leu314 (PCL-1) to Ile54 (PHO85-1) during 81.72% of time.

### PCL-1 influences the germination rate and cell cycle progression and localizes in different cellular compartments

**FIGURE 5:**
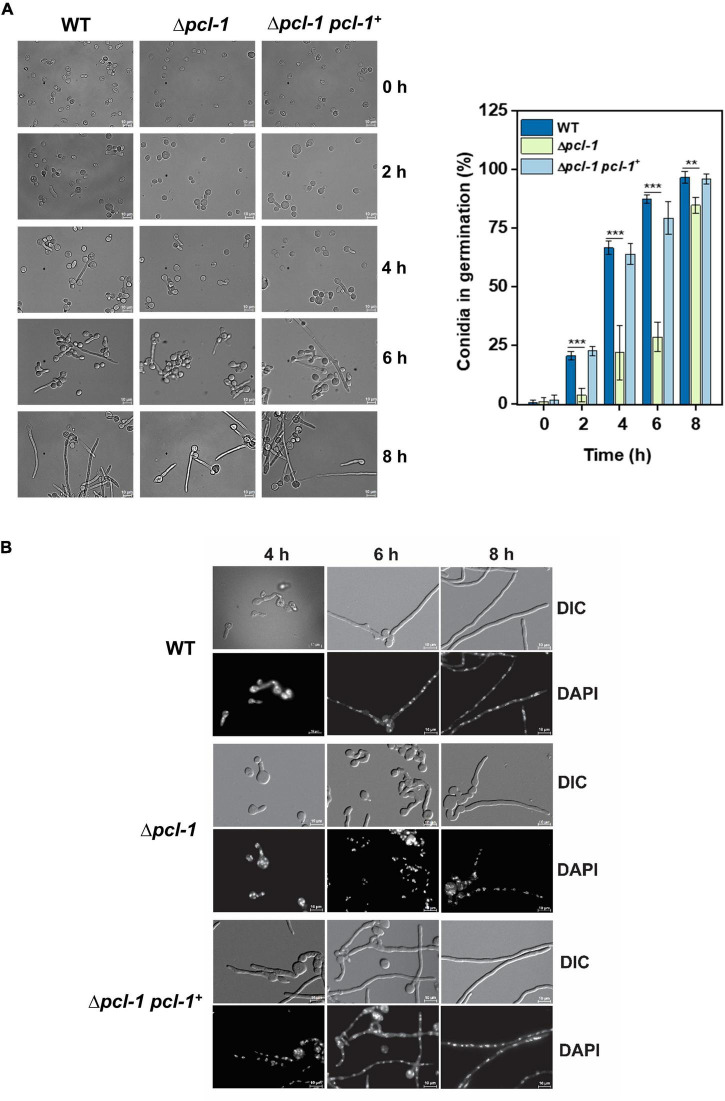
Δ*pcl*-1 strain shows defective timing in germination. For germination analysis, conidia from wild-type, Δ*pcl-1* mutant, and Δ*pcl-1 pcl-1^+^* complemented strains were inoculated into VM medium, and aliquots were taken at the indicated time points for microscopic assay. **(A)** Conidia germination in submerged cultures at 150 rpm at different time points. Images were captured using an AXIO Imager.A2 Zeiss microscope, at a magnification of 630 X. The results were expressed as percentage of conidia in germination (right panel) and represent the standard deviations from three independent experiments. **(B)** Conidia germinated on coverslip were fixed with formaldehyde in PBS, the nuclei were stained with DAPI, and the fluorescence was analyzed using the microscope AXIO Imager.A2 (Zeiss) coupled to AxioCam camera at a magnification of 1000 X. *t*-test was used for the *P*-values. **P < 0.001; ***P < 0.0001.

As *N. crassa* synthesizes a limited number of cyclins (Borkovich et al., 2004; Hong et al., 2014), and that in yeast different cyclins direct the Pho85p kinase to different functions, we decided to investigate whether PCL-1 is involved in additional cellular functions. To verify whether PCL-1 influenced growth, we first analyzed conidia germination of the WT and Δ*pcl* strains and the Δ*pcl-1 pcl-1^+^* complemented strain in samples collected from liquid cultures under agitation. The amount of conidia in germination was quantified at different times and we observed a delay in the germination rate of the Δ*pcl* strain at the initial times of culture, mainly up to 6 h of growth. However, normal growth rate was achieved at later times of culture (8 h) (Figure 5A, left and right panels). Conidia germination was also analyzed in conidia inoculated onto coverslips and covered with liquid medium (Figure 5B). In this assay, the delay in germination is more evident because the mutant strain shows shorter hypha, mainly at the time of 6 h, when compared to the wild-type strain. The germination delay was not observed in the complemented strain indicating that the phenotype was due to the PCL-1 lacking. It is important to mention that no changes in the hypha morphology was observed, and that the complemented strain expresses the protein under the control of the native promoter.

**FIGURE 6:**
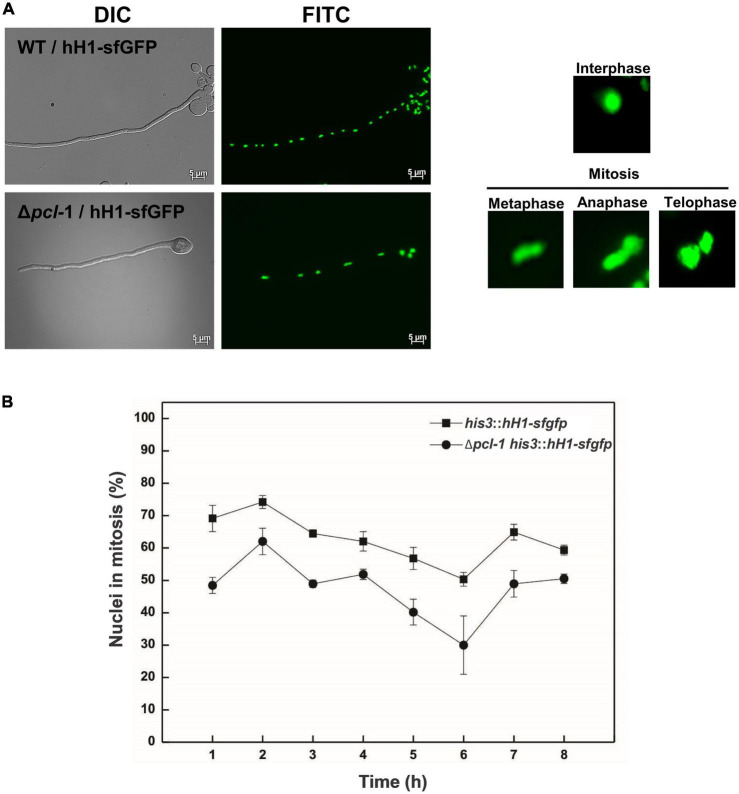
Δ*pcl*-1 strain shows lower number of mitotic nuclei compared to the wild-type strain. For cell division analysis, conidia from Δ*pcl*-1 *hH1-sfgfp* and hH1-*sfgfp* strains were germinated in liquid VM medium at the indicated time points. **(A)** General view of a hypha from each strain (left panel) and the nuclei in the different mitosis phases used for quantification (right panel). **(B)** Aliquots were removed during germination, and the fluorescence was analyzed. Six images were taken from each time point and the nuclei undergoing mitosis were counted. The result is representative of three independent experiments.

To investigate the PCL-1 involvement in cell cycle progression, we constructed the Δ*pcl-1 hH1-sfgfp* strain, a Δ*pcl-1* mutant strain that synthesizes the sfGFP-fused H1 histone (Figure 6A) and allows quantification of the nuclei in different mitotic phases (Figure 6A, right panel). Fewer nuclei under division were counted in the mutant strain compared to the non-mutant strain (hH1-sfGFP) indicating that PCL-1 may also control cell cycle progression (Figure 6B). We have not observed accumulation of cells in any specific mitotic phase, suggesting that PCL-1 is continuously synthesized during the cell cycle. The germination and cell progression data suggested that PCL-1 may play a role in fundamental cellular processes.

**FIGURE 7:**
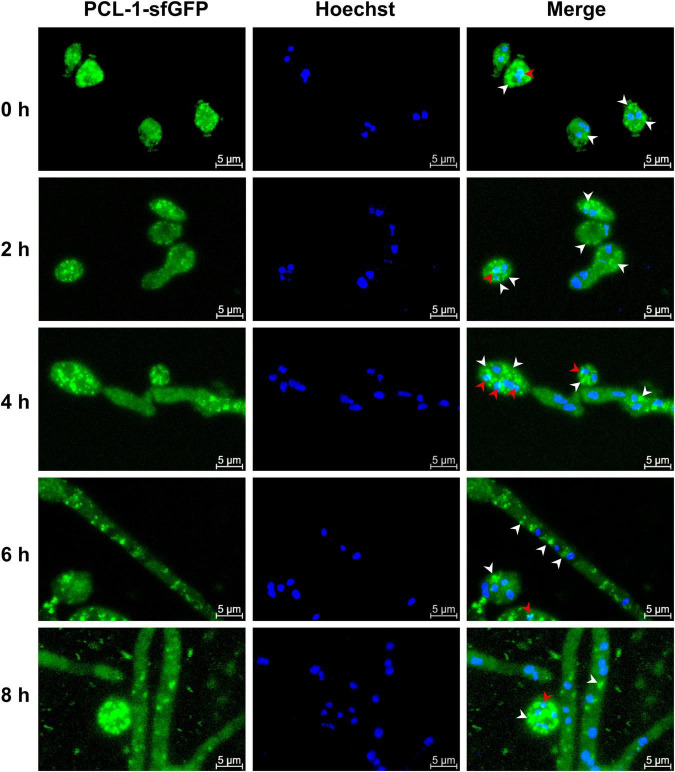
PCL-1-sfGFP cyclin locates in the cytoplasm and nucleus. Conidia from the Δ*pcl-1 pcl-1*^+^ complemented strain were grown on coverslips in liquid VM medium, and the fluorescence was evaluated at the indicated time points. Cells were fixed in PBS with formaldehyde, the nuclei were stained with Hoechst (10 μg per mL), and the fluorescence was evaluated in a Confocal Laser Microscopy. The images are representative of at least three independent experiments. Protein showing cytoplasm (white arrow) and nucleus (red arrow) location.

Since PCL-1 is involved in different cellular processes, we next investigated the cellular distribution pf PCL-1 at different times of conidia germination using the Δ*pcl-1 pcl-1^+^* complemented strain, which express sfGFP-fused PCL-1. The conidia of the strain showed a strong fluorescent signal throughout the cytoplasm (Figure 7, 0 h). Interestingly, during germination, cells displayed a noticeably subcellular accumulation of the protein in the cytosol of hyphae (Figure 7, later times, white arrowheads) indicating that PCL-1 may accumulate in certain cell regions during germination as granular clusters. In addition, accumulation in nuclei was also observed (Figure 7, red arrowheads), suggesting that the protein may also translocate into nucleus, although classical nuclear localization signals (NLS) were not identified in the protein primary sequence.

### PCL-1 plays a role in the regulation of calcium metabolism and influences the expression and localization of the CRZ-1 transcription factor

**FIGURE 8:**
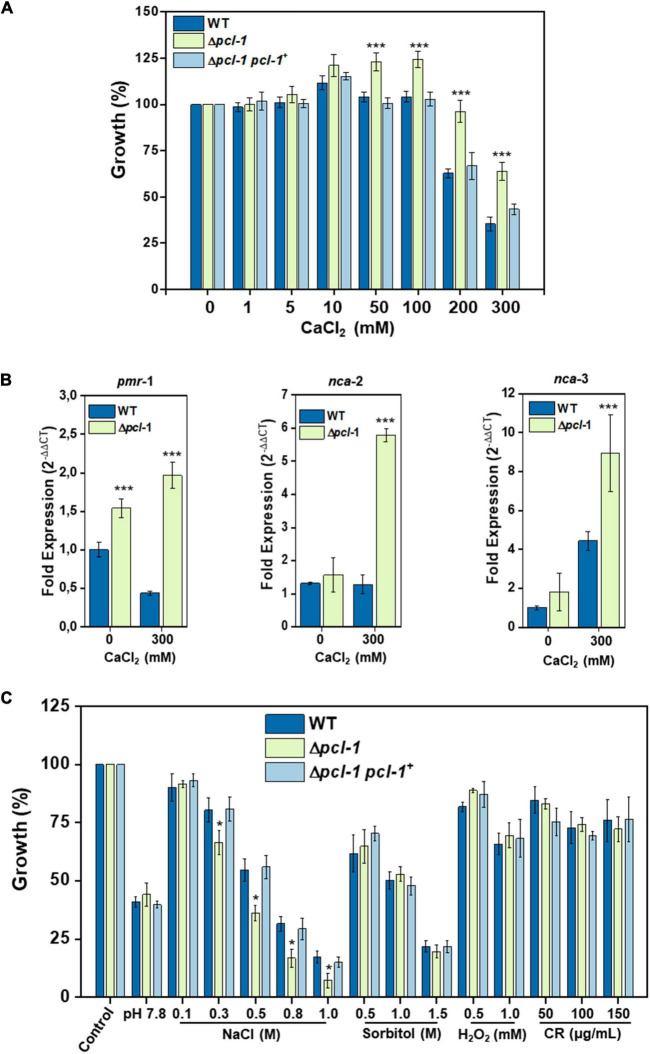
PCL-1 is involved in calcium stress response. Growth of the wild-type, Δ*pcl*-*1*, and Δ*pcl*-*1 pcl*-*1*^+^ complemented strains under different stressing conditions. **(A)** Growth in the presence of calcium. Strains were grown in solid VM medium containing increased calcium chloride concentration. Growth was evaluated after 24 h and is expressed as percentage of growth in the absence of calcium. **(B)** Expression of calcium responsive genes. Strains were grown in the presence and absence of 300 mM calcium for 24 h and total RNA was extracted. Expression of *pmr*-1, *nca*-2, and *nca*-3 genes was assayed by qPCR using the *tub*-2 gene as reference gene. C_t_ values were used to estimate the log2^– ΔΔCt^ fold difference between control (without calcium) and growth in the presence of 300 mM calcium. **(C)** Strains growth in the presence of different stressors agents. Strains were grown in solid VM media, and increased concentration of the stress agents. Growth was evaluated after 24 h and is expressed as percentage of growth in the absence of stress. Error bars represent the standard deviations from three independent experiments. *t*-test was used for the *P*-values. *P < 0.005; ***P < 0.0001.

In *S. cerevisiae*, the Pho85 protein kinase, together with Pho80 partner cyclin, is a kinase that phosphorylates the zinc finger Crz1 transcription factor, which is involved in the regulation of calcium metabolism and activated by dephosphorylation by the calcineurin phosphatase (Carroll and O’Shea, 2002; Sopko et al., 2006). The fact that *N. crassa* synthesizes low number of cyclins (Borkovich et al., 2004), prompted us to investigate if PCL-1 could be a cyclin involved in calcium metabolism regulation. Hyphal growth in Petri dishes of the Δ*pcl-1* strain was strongly increased under high calcium chloride concentration (200–300 mM) suggesting that PCL-1 participates in the calcium stress response (Figure 8A; Supplementary Figure 3). The Δ*pcl-1 pcl-1^+^* complemented strain exhibited comparable growth to the wild-type strain (Figure 8A; Supplementary Figure 3), confirming that the growth defect in the mutant strain was indeed due to the *pcl-1* deletion.

The expression of three genes encoding Ca^2++^-ATPases, *pmr-1* (NCU03292), *nca-2* (NCU04736), and *nca-3* (NCU05154) (Bowman et al., 2011, 2012) was analyzed after growing the wild-type and mutant strain under 300 mM calcium chloride for 24 h. Gene expression of all three genes was increased in the mutant strain compared to the wild-type strain indicating a role for PCL-1 in the expression regulation of these genes and, therefore, in the control of the calcium stress response in *N. crassa* (Figure 8B). To investigate if PCL-1 influences the cellular response to other stressors, we analyzed growth of the wild-type, mutant, and complemented strains in VM pH 7.8 (alkaline pH stress) and in VM containing NaCl and sorbitol (osmotic stress), H_2_O_2_ (oxidative stress), and Congo Red (plasma membrane/cell wall stress). The mutant strain showed sensitivity only to osmotic stress induced by NaCl (Figure 8C).

**FIGURE 9:**
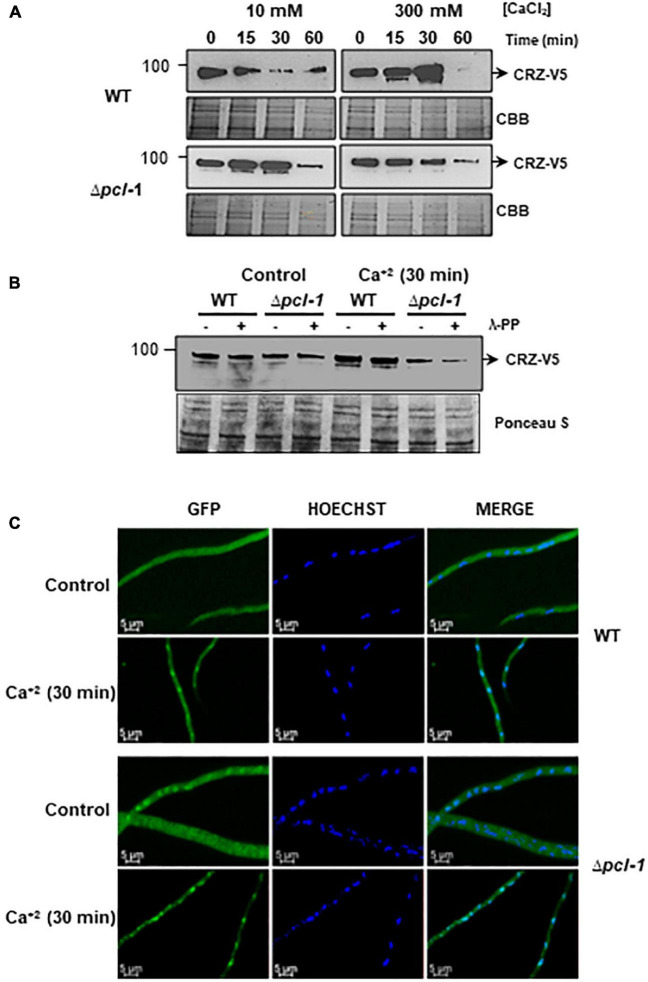
CRZ-1 expression and cellular localization are modulated by PCL-1. **(A)** CRZ-1-V5 expression was analyzed in cellular extracts from wild-type and Δ*pcl-1* strains expressing V5-tagged CRZ-1 under stress induced by either 10 or 300 mM calcium chloride up to 1 h. 30 μg of total protein treated with 400 U λ-phosphatase was assayed in 8% SDS-PAGE. The blot represents a typical result from two biological replicates. CBB, gel stained with Coomassie brilliant blue used as loading control. **(B)** CRZ-1-V5 phosphorylation assay in 4–15% Mini-PROTEAN^®^ TGX™ Precast Protein Gels using 30 μg total protein from crude cell extracts incubated or not with 300 mM calcium chloride for 30 min and treated or not with λ-protein phosphatase. Proteins were electrotransferred to a nitrocellulose membrane and probed with the anti-V5 monoclonal antibody (Invitrogen, Waltham, USA). The same gel stained with Ponceau S is shown as loading control. The numbers on the left side represent the MW in kDa. **(C)** Cellular localization of CRZ-1-GFP is modulated by PCL-1. Wild type and Δ*pcl-1* strains expressing CRZ-1-GFP were grown in coverslips in VM medium for 24 h and subjected to calcium stress for 30 min. A control sample without calcium was used as a control. Cells were fixed in PBS with formaldehyde, the nuclei were stained with Hoechst (10 μg per mL), and the fluorescence was evaluated in a Confocal Laser Microscopy. The images are representative of at least three independent experiments.

As the mutant strain exhibited increased growth and upregulation of calcium responsive genes under high calcium chloride concentration, we decided to extend the investigation on the calcium-responsive CRZ-1 transcription factor. We first analyzed the protein expression in the wild-type and Δ*pcl-1* strains both endogenously expressing C-terminus V5-tagged CRZ-1 at different times of growth under low (10 mM) and high (300 mM) calcium chloride concentrations. In this experiment, the protein samples were treated with phosphatase due to the high number of putative phosphorylation sites that might exist in the *N. crassa* protein. CRZ-1-V5 migrates as an apparent molecular mass between 90 and 100 kDa. Interestingly, we observed variation in the CRZ-1 expression both in a strain- and calcium chloride dependent manner (Figure 9A). Under high calcium chloride concentration the CRZ-1 expression in the wild-type strain increased in the time range from 0 to 30 min compared with low concentration. However, very low levels of protein were detected at 60 min under the same condition. On the other hand, the expression in the Δ*pcl-1* strain decreased under high calcium chloride concentration in the time range from 0 to 60 min compared with low calcium concentration. In summary, CRZ-1 expression is modulated by calcium concentration and by PCL-1.

It was previously mentioned here that the *S. cerevisiae* Pho85 kinase, together with Pho80 cyclin, is a kinase that phosphorylates and inactivates the Crz1p transcription factor (Sopko et al., 2006). Phosphorylated Crz1p localizes in the cytosol (Cyert, 2003). Based on the multiple functionalities of PCL-1, we hypothesized that CRZ-1 would be less phosphorylated in the Δ*pcl-1* strain than in the wild-type strain, and therefore, being able to be translocated to the nucleus. The *A. fumigatus* CrzA orthologous transcription factor has been reported as a highly phosphorylated protein *in vivo*, and a total of twenty phosphorylation sites were identified in the CrzA by mass spectroscopy; some of them predicted as potential target motifs for CDKs by bioinformatic tool (Shwab et al., 2019). Comparison between the *A. fumigatus* CrzA and the *N. crassa* CRZ-1 proteins by sequence alignment (Supplementary Figure 4) showed that many putative phosphorylation sites are conserved in the *N. crassa* protein (highlighted in red), including two potential CDK targets. We decided to analyze the phosphorylation status of CRZ-1-V5 in the wild-type and Δ*pcl-1* strains under high (300 mM) calcium chloride concentration (Figure 9B). Again, we observed in the mutant strain lower CRZ-1 expression than the expression in the wild-type strain in the presence of calcium. In addition, both strains exhibited similar expression levels in the absence of calcium (control, compare to Figure 9A at 0 time). However, we did not observe any differences in the phosphorylation levels of CRZ-1, even after protein phosphatase treatment. Using PhosTag gel as a different experimental approach to find out whether phosphorylation of CRZ-1 is affected by PCL-1 we were not able obtain a conclusive result, most likely due to the many putative phosphorylation sites in CRZ-1 (results not shown).

We decided to analyze the CRZ-1-GFP cellular localization in the wild-type and Δ*pcl-1* background strains, both expressing the protein under the control of the native promoter, in the presence of calcium chloride (300 mM) (Figure 9C). In the wild-type strain, strong fluorescence was visualized throughout the cytoplasm in the absence of calcium, as expected (Figure 9C, control). However, upon calcium stress, the protein translocates into nucleus (Figure 9C), as described for the *A. fumigatus* and *A. nidulans* CrzA orthologs (Soriani et al., 2008; Hernández-Ortiz and Espeso, 2013). Interestingly, in the Δ*pcl-1* strain, in the absence of calcium we observed protein localization both in the nuclei and cytoplasm, depending on the hyphae analyzed. On the other hand, upon calcium stress, the protein mainly localized in the nuclei (Figure 9C). These results strongly support our hypothesis, in which CRZ-1 is less phosphorylated in the Δ*pcl-1* background strain compared to the wild-type background strain, being able to translocate into nucleus and to regulate the expression of calcium responsive genes.

## Discussion

Cyclins are defined as proteins that are periodically synthesized and degraded in every mitosis, and although characterized as regulatory partners for CDKs they also play CDK-independent cellular functions. The multinucleated fungi *Neurospora crassa* is described as having a few cyclins likely involved in cell cycle control (Borkovich et al., 2004), some of them showing circadian oscillations indicating a connection between cell cycle and circadian rhythms (Hong et al., 2014; Zámborszky et al., 2014). Because of the repeat-induced point mutation (RIP) mechanism (Selker, 2002), it is expected the existence of non-redundant and, therefore, less specialized cyclins in Neurospora compared to yeasts and higher eukaryotes. In this work, a new *N. crassa* cyclin was identified in a search for a PHO85-1 partner cyclin, a protein kinase orthologous to the *Saccharomyces cerevisiae* Pho85 kinase that together with Pcls from the Pho80 subfamily regulates glycogen metabolism by phosphorylating glycogen synthase, the rate limiting enzyme in glycogen synthesis (Huang et al., 1996, 1998). The protein was named as PCL-1, and the presence of a typical cyclin box domain (CBD) (Tatum and Endicott, 2020) predicted by AlphaFold confirms that PCL-1 is a real cyclin that performs functions encompassing different *S. cerevisiae* Pcls families.

Mammalian cells possess a complex network of cyclins and CDKs to ensure an accurate progression of cell cycle, having different cyclin/CDK complexes specific for each phase of the cell cycle. On the other hand, in yeast cells, only one CDK, Cdc28 in *S. cerevisiae* and Cdc2 in *Schizosaccharomyces pombe*, controls the entire cell cycle (Harashima et al., 2013; Lim and Kaldis, 2013), and in *N. crassa*, like yeasts, only one CDK (CDC-2) participates in the cell cycle progression. Regarding cyclins, only three cyclins likely involved in cell cycle progression are described in *N. crassa* (Borkovich et al., 2004; Kuwabara et al., 2020) suggesting that the CDK/cyclin complexes are more simplified compared to the yeast models. In this work, we identified a multifunctional cyclin that together with PHO85-1 CDK regulates glycogen metabolism by phosphorylating glycogen synthase (GSN).

PCL-1 plays a role in cell cycle progression and in germination indicating that, complexed with a CDK, the heterodimer translocates into nucleus and phosphorylates several target proteins inducing cell-cycle regulation. Although we have not identified a classical nuclear localization signal (NLS), a common characteristic described in CDK/cyclins complexes (Moore et al., 1999; Porter and Donoghue, 2003), we observed PCL-1 localization both in nucleus and cytoplasm during germination indicating that it requires a protein partner to be translocated into nucleus. Little is known concerning cell cycle regulators and their nucleocytoplasmic trafficking in filamentous fungi. While yeasts are mononucleated cells, Neurospora is a multinucleate organism having in the cytoplasm nuclei in different phases of the cell cycle. Therefore, the nuclei divide asynchronously, and their cell cycles must be regulated independently (Serna and Stadler, 1978), which may explain our results regarding the protein localization both in nucleus and cytoplasm. A recent study demonstrated that each nucleus selects a special cyclin only when necessary, maintaining nuclei in different cell cycle phases in the same cytoplasm (Kuwabara et al., 2020).

Beyond cell cycle control, CDKs and cyclin members have been described to influence various cellular processes, including metabolism, independently of their partners (Lim and Kaldis, 2013). In *S. cerevisiae* cells, the accumulation of glycogen and trehalose also depends on the Cdc28 activity (Zhao et al., 2016). We demonstrated here that PCL-1 participates in the regulation of glycogen metabolism. The Δ*pcl-1* strain accumulates higher glycogen levels and exhibits higher glycogen synthase activity ratio (−/+ G6P, Figure 2B) than the wild-type strain, features that imply PCL-1 as a cyclin involved in the control of the glycogen metabolism in *N. crassa*. In addition, the existence of less phosphorylated glycogen synthase isoforms in the mutant strain supports its role in the glycogen synthase phosphorylation and, therefore, in the maintenance of proper glycogen levels. By producing recombinant PCL-1 cyclin and PHO85-1 CDK in *E. coli*, we were able to reconstitute the functional PHO85-1/PCL-1 complex and to demonstrate that the Ser636 is the amino acid residue *in vitro* phosphorylated by the complex, demonstrating that PCL-1 is required for phosphorylation of GSN by the PHO85-1 kinase. By sequence alignment this residue may correspond to Ser654 in the *S. cerevisiae* Gsy2p (Figure 3A), which together with Thr667 are both *in vitro* and *in vivo* phosphorylated by the Pho85p/Pcl10p complex (Huang et al., 1996; Wilson et al., 1999). Therefore, two from the three yeast Gsy2p phosphorylation sites are phosphorylated by this protein complex. Surprisingly, the *N. crassa* PHO85-1/PCL-1 complex is not able to phosphorylate the Thr645 residue in GSN, which may resemble to the yeast Thr667 residue, and does not phosphorylate none of the additional putative sites. Our results regarding GSN phosphorylation are very interesting and differ from the more extensively characterized Gsy2p phosphorylation. Only one amino acid residue is phosphorylated by the PHO85-1/PCL-1 complex, which implies that additional kinases, different from CDKs, may be required for phosphorylation of the other three putative sites. It is important to mention that various protein kinases are described to phosphorylate the nine sites existent in the muscle glycogen synthase (Roach et al., 2012). Like the yeast Pho85p kinase, PHO85-1 does not appear to require phosphorylation for activation, as required for others CDKs, since it is a *E. coli* recombinant protein.

The structural prediction of the PHO85-1 monomer by AlphaFold revealed regions such as PSTAIRE and T-loop with low confidence, however, a high-confidence model was obtained when PHO85-1 was predicted in complex to its partner PCL-1 (Supplementary Figure 2). PHO85-1 models predicted by AlphaFold suggest intermediate conformations that can highlight the activation process of CDKs, observed by structural modifications mainly caused by the PSTAIRE helix rotation and further salt-bridge formation between Lys39 and Glu56 residues when bound to cyclins. Such movements are well-known in CDKs, which are required for cyclin binding (De Vivo et al., 2011). The interface of the complex represents a common CDK/cyclin dimer (Zheng and Quiocho, 2013; Wood and Endicott, 2018; Tatum and Endicott, 2020) with PSTAIRE playing a central role in dimer stabilization. MD simulations of the complex showed that PSTAIRE and T-loop residues are essential for PCL-1 recognition and stabilization, described mainly by a salt-bridge between an arginine-rich region of PHO85-1 and Asp280 of PCL-1 (Figure 4A). This region constitutes the so-called Substrate Recognition Segment (SRS) with such interactions being observed by Arg132 and Asp376 in PHO85/Pcl10 (Zheng and Quiocho, 2013). In other complexes, these arginines are interacting with mainchain oxygens of cyclin residues, such as Phe267, Glu269, and Ile270, as observed in the CDK2/Cyclin A complex (Jeffrey et al., 1995). In summary, the modeling study of the *N. crassa* PHO85-1/PCL-1 complex revealed these proteins as a classical CDK/cyclin complex. The importance of the PSTAIRE helix of PHO85-1 in complex stabilization is shown, as well as the structural particularities of the SRS in comparison to other CDK/cyclin complexes.

We also describe here that PCL-1 regulates calcium metabolism in *N. crassa*; Δ*pcl-1* cells grow better than the wild-type strain and calcium responsive genes are overexpressed in this strain under high calcium concentration. The Calcineurin-Responsive-Zinc-fingers (CRZ) in the fungal kingdom is the main regulator in the calcium stress response. It is described as a highly phosphorylated protein in *A. fumigatus* (Shwab et al., 2019), and several proteins kinase were identified to phosphorylate Crz1p in yeast, among them the Pho85p/Pho80p CDK/cyclin complex (Sopko et al., 2006; Hernández-Ortiz and Espeso, 2017). Upon calcium stress, calcineurin dephosphorylates CRZ causing its translocation from cytosol into nucleus triggering expression of calcium responsive genes (Stathopoulos-Gerontides et al., 1999). We asked whether PCL-1 could regulate the CRZ-1 phosphorylation status and, therefore, to influence its cellular location. Although we were not able to observe changes in the CRZ-1 phosphorylation in Δ*pcl-1* cells expressing V5-tagged CRZ-1, we did observe nuclei fluorescence in certain hypha synthesizing GFP-tagged CRZ-1, suggesting the presence of less phosphorylated CRZ-1-GFP in Δ*pcl-1* cells in the absence of calcium. We conclude that CRZ-1 nuclear localization induces expression of calcium responsive genes allowing better growth of Δ*pcl-1* cells under calcium stress, as we demonstrate here. An intriguing result reported here concerns the regulation of CRZ-1 expression dependent on calcium concentration, and the control by PCL-1 may represent additional information regarding a new manner of CRZ-1 activity modulation. In *S. cerevisiae*, the abundance of Crz1-GFP was described to remain constant over a few hours in the presence of calcium (Cai et al., 2008).

**FIGURE 10:**
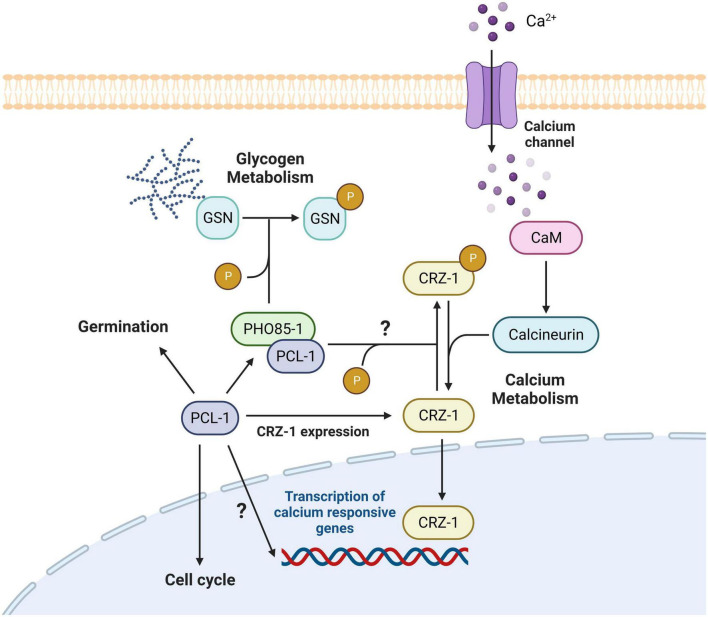
Proposed model for the multifunctional role of the PCL-1 cyclin in *N. crassa.* PCL-1 is involved in conidial germination since Δ*pcl-1* cells shows reduced germination at the initial germination times and in cell cycle progression since Δ*pcl-1* cells presents less nuclei in mitotic phases. PCL-1 together PHO85-1 protein kinase regulates glycogen metabolism by directly *in vitro* phosphorylating glycogen synthase (GSN) at the Ser636 amino acid residue. As consequence, in Δ*pcl-1* cells GSN is more active and the levels of glycogen are higher compared to the wild-type cells. Moreover, PCL-1 is involved in calcium stress response. Δ*pcl-1* cells exhibit higher growth than the wild-type cells in the presence of high calcium chloride concentration by overexpressing calcium regulated genes. In addition, the CRZ-1 transcription factor also locates in nuclei in these cells supporting our hypothesis that CRZ-1 is less phosphorylated in the Δ*pcl-1* strain being able to translocate into nucleus. Created by BioRender.com.

In this work, we have characterized a new cyclin in *N. crassa* that participates in multicellular events besides being a cyclin involved in cell cycle control and progression (Figure 10). PCL-1 is an activator of the PHO85-1 CDK in the glycogen metabolism regulation leading to higher GSN phosphorylation (Figure 10). In addition, PCL-1 influences the CRZ-1 expression and its phosphorylation, which modulates its cellular location. Although we have not identified the protein kinase acting in the CRZ-1 modulation, we suppose that PHO85-1 may act as the PCL-1 partner based on what is described for *S. cerevisiae*. These observations extend our understanding concerning cyclins’ role and how cyclins may mediate their functions in a multinucleated cell such as a filamentous fungus.

## Data availability statement

The original contributions presented in this study are included in this article/Supplementary material, further inquiries can be directed to the corresponding author.

## Author contributions

JC, TC, LB, CL, and EH performed the experiments. AG carried out the protein modeling experiments under supervision of MF. PB helped in the confocal microscopy. MB designed the work and wrote the manuscript with contribution of JC and AG. All authors approved the submitted version.

## Footnotes

1
http://www.fgsc.net

2
http://www.fgsc.net/Neurospora/NeurosporaProtocolGuide.htm

3
https://doi.org/10.1038/s41586-021-03819-2

4
https://doi.org/10.1101/2021.10.04.463034

5
https://doi.org/10.1074/jbc.M113.502773

6
http://fungidb.org
