# Supplementary material for: The Neurospora crassa PCL-1 cyclin is a PHO85-1 (PGOV) kinase partner that directs the complex to glycogen metabolism and is involved in calcium metabolism regulation
Source: Front Microbiol. 2022 Dec 22;13:1078972. doi: 10.3389/fmicb.2022.1078972 (PMC9815767; doi:10.3389/fmicb.2022.1078972)
Supplement: Supplementary file 1 [file Data_Sheet_1.PDF]

SUPPLEMENTARY MATERIAL

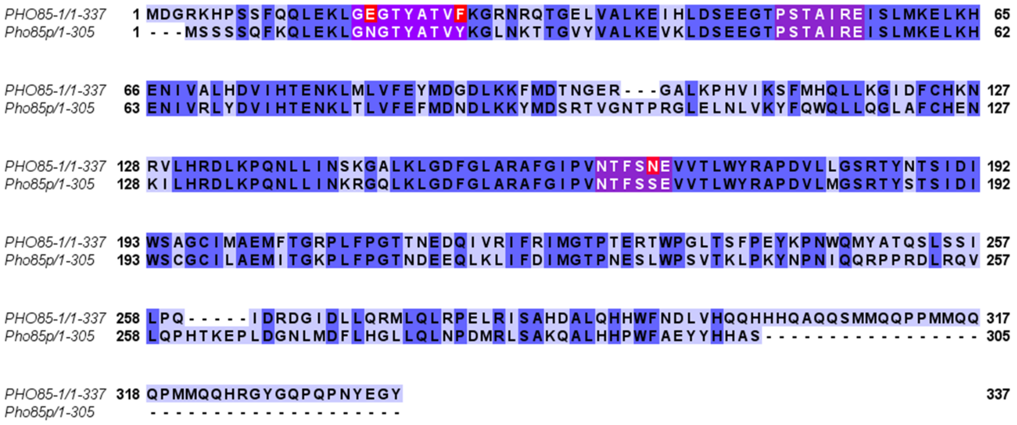

Fig. S1. Sequence alignment between the *N. crassa* PHO85-1 and *S. cerevisiae* Pho85p proteins**.** Alignment was performed using ClustalW2, and the identical (dark blue) and conservative (light blue) amino acid residues were identified using Jalview Version 2 (Waterhouse et al., 2009). The classical CDK domains PSTAIRE-like and glycine-rich, and the T-loop region are shown in purple, and the non-conservative residues in these domains are shown in red.

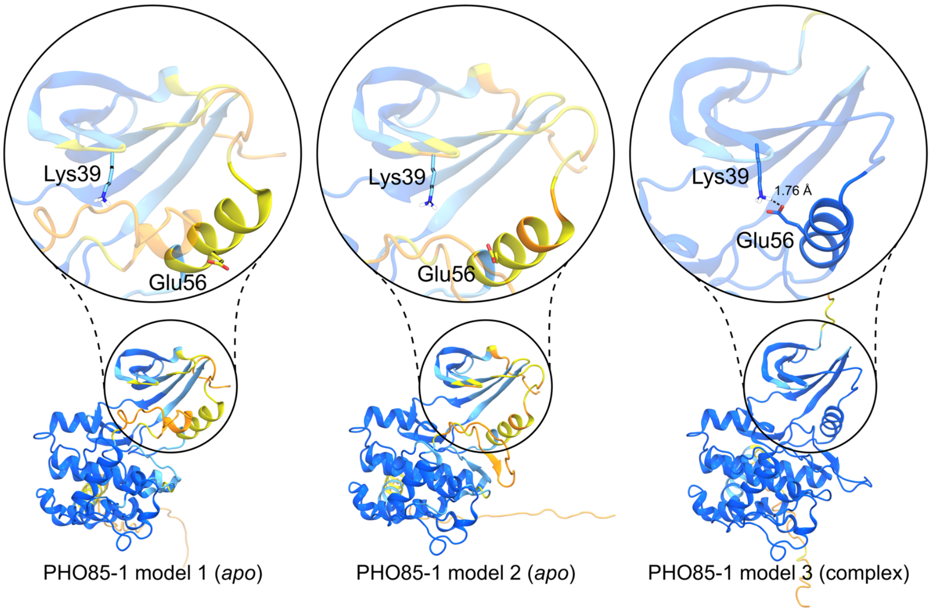

Fig. S2. PHO85-1 model prediction by AlphaFold. Two *apo* conformations (model 1 and model 2) diverged according to PSTAIRE and T-loop orientation, which presented low (yellow) or very low (orange) model confidence. On the other hand, when PHO85-1 was predicted in complex to PCL-1 (not shown) such regions presented considerable model confidence, shown in blue or cyan. When bound to PCL-1, PHO85-1 presents a salt-bridge formation between Lys39 and Glu56 residues (zoom-in-view).

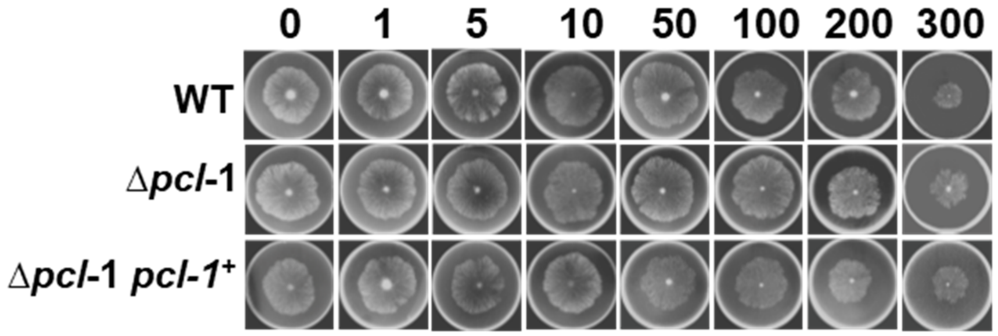

Fig. S3. Δ*pcl*-1 cells exhibit tolerance to high calcium chloride concentration in plates. Conidia from the wild-type, Δ*pcl*-1, and Δ*pcl*-1 *pcl*-1$^+$ strains were inoculated into plates containing solid VM with 2% sucrose and increased amounts of calcium chloride (0 to 300 mM). Cells were grown for 24 h at 30 °C. Images of the plates were acquired using the ChemiDoc Image System (BioRad).

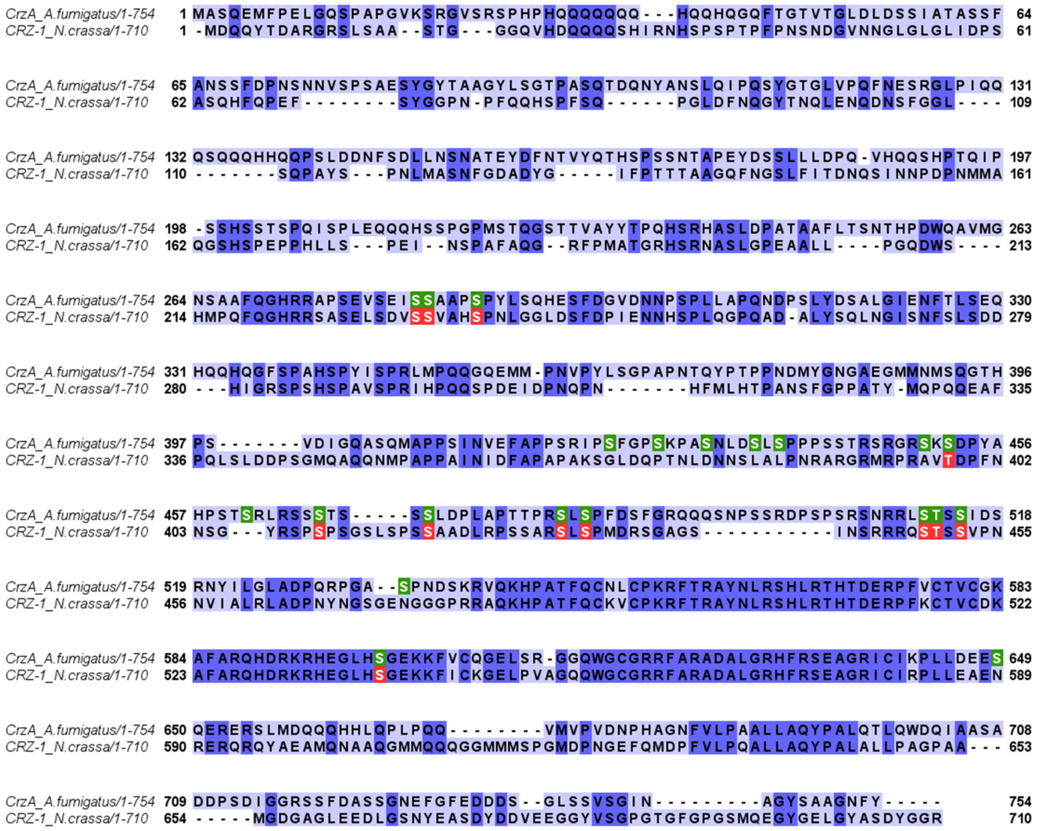

Fig. S4. Sequence alignment between the *N. crassa* CRZ-1 and the *A. fumigatus* Crz1 proteins. Alignment was performed with ClustalW2 and the identical (dark blue) and conservative (light blue) amino acid residues were identified with Jalview Version 2 (Waterhouse et al., 2009). The putative phosphorylating sites identified in the Crz1 protein are shown in green (Shwab et al., 2019) and the conservative putative phosphorylating sites in CRZ-1 are shown in red.

Table S1. Oligonucleotides used in this work.

Primers	Sequences (5'→3')*	Source
**pET28a-*pcl*-1 c DNA cloning**		
Nc8772-F	CAT**ATG**TCACCCAATCCCGAACAC	NCU08772
Nc8772-R	GGATCC**TCA**CGCCTCGGCGGCAGCTTC	NCU08772
**pET28a-*pho85*-1 cDNA cloning**		
Nc7580-F	GTACAT**ATG**GACGGCAGGAAACACCC	NCU07580
Nc7580-R	CGGGATCC**CTA**GTAGCCCTCGTAGTTG	NCU07580
**pETsumo-*gsn* cDNA cloning**		
GSN-*Bam*HI-F	GGCGGATCCATGGCCCACGACAACCG	PET28a-*gsn***
GSN-*Eco*RI-R	GCGAATTCTTACTCGACTCCTGGAAT	PET28a-*gsn*
**pTSL91A-*pcl-1-sfgfp* construction and Δ*pcl-1* complementation**		
8772sGFP-F	CTAGTCTAGACAAAAC**ATG**TCACCCAATCCCGAAC	NCU08772
8772sGFP-R	CCGCTTAATTAAtcctcctcctcctcctccCGCCTCGGCGGCAGCTTCC	NCU08772
np8772-F	ATAAGAATGCGGCCGCTGCAACAGGCTGGGGAGGG	P*pcl*-1
np8772-R	CTAGTCTAGAGTTGGATATGGAATTCGATTC	P*pcl*-1
**GSN mutagenesis*****		
S632A-F	AAGATATCTCGTCCCTTC**G**CAGTACCGGGCTCGCC	PET28a-*gsn*
S632A-R	GGCGAGCCCGGTACTG**C**GAAGGGACGAGATATC	PET28a-*gsn*
S636A-F	CCCTTCTCAGTACCGGGC**G**CGCCTAGAGACCGGAC	PET28a-*gsn*
S636A-R	GTCCGGTCTCTAGGCG**C**GCCCGGTACTGAGAAGGG	PET28a-*gsn*
T641A-F	CGCCTAGAGACCGG**G**CTGGTATGATGACCCCTG	PET28a-*gsn*
T641A-R	CAGGGGTCATCATACCAG**C**CCGGTCTCTAGGCG	PET28a-*gsn*
T645A-F	GACCGGACTGGTATGATG**G**CCCCTGGCGATTTTGC	PET28a-*gsn*
T645A-R	GCAAAATCGCCAGGGG**C**CATCATACCAGTCCGGTC	PET28a-*gsn*
S636ADM-F	CAGTACCGGGC**G**CGCCTAGAGACC	PET28a-*gsn*
S636ADM-R	GGTCTCTAGGCG**C**GCCCGGTACTG	PET28a-*gsn*
T645ADM-F	CTGGTATGATG**G**CCCCTGGCGATTTTG	PET28a-*gsn*
T645ADM-R	CAAAATCGCCAGGGG**C**CATCATACCAG	PET28a-*gsn*
**RT-qPCR**		
pmr-1-F	GCTCGGACTCCTTGATTTG	NCU03292
pmr-1-R	ACACCCATATGCCTCCTT	NCU03292
nca-2-F	GCCTGTTCCCTCCTAAGT	NCU04736
nca-2-R	TCTCCTGCTCCTCATTGAT	NCU04736
nca-3-F	AAGAAGAAGGAGGGAGAGG	NCU05154
nca-3-R	CCTTGAACTATGCACTCTCG	NCU05154
tub-2-F	CCATGTACCCTGGTCTCTCCGAC	NCU04054
tub-2-R	CCACCGATCCAGACGGAGTACTTG	NCU04054
**CRZ-1-GFP/V5 strains construction**		
CrzGLY-F	GATCAGCCCACTAACCTGGA	NCU07952
CrzGLY-R	cctccgcctccgcctccgccgcctccgccACGGCCGCCGTAGTCACTC	NCU07952
CrzLox-F	tgctatacgaagttatggatccgagctcgGCATAGTGTCATGACATTTC	NCU07952
CrzLox-R	GGTCTGCGAAAAGGTGGTAG	NCU07952
10Gly-F	GGCGGAGGCGGCGGAGGCGGAGGCGGAGG	pZERO-V5/GFP
LoxP-R	TGCTATACGAAGTTATGGATCCGAGCTCG	pZERO-V5/GFP
Hph-F	AAAAAGCCTGAACTCACCGCGACG	pZERO-V5/GFP
Hph-R	GGTCATTGACTGGAGCGAGGCGA	pZERO-V5/GFP
V5-R	CGGGGTACCTCACGTAGAATCGAGACCGAG	V5 Tag
GFP-R	CTTGTACAGCTCGTCCATGCCG	GFP tag

*The start and stop codons are shown in bold. The restriction sites are underlined (*Nde*I/*Bam*Hi – Nc8772-F/R, *Nde*I/*Bam*HI – Nc7580-F/R, *Pac*I/*Xba*I – 8772sGFP-F/R, *Not*I/*Xba*I – np8772-F/R) in the oligonucleotides. The nucleotide sequences encoding either 6Gly or LoxP are represented in lower cases. ORFs NCU08772 (*pcl-1*), NCU07580 (*pho85-1*), NCU06687 (*gsn*, glycogen synthase) and NCU07952 (*crz-*

1). **pET28a-*gsn* (55). ***The mutagenesis sites are in bold in the oligonucleotides.
